# High-Efficiency FLP and ΦC31 Site-Specific Recombination in Mammalian Cells

**DOI:** 10.1371/journal.pone.0000162

**Published:** 2007-01-17

**Authors:** Christopher S. Raymond, Philippe Soriano

**Affiliations:** Program in Developmental Biology, Division of Basic Sciences, Fred Hutchinson Cancer Research Center, Seattle, Washington, United States of America; UMass Medical School, United States of America

## Abstract

DNA site-specific recombinases (SSRs) such as Cre, FLPe, and φC31, are powerful tools for analyzing gene function in vertebrates. While the availability of multiple high-efficiency SSRs would facilitate a wide array of genomic engineering possibilities, efficient recombination in mammalian cells has only been observed with Cre recombinase. Here we report the *de novo* synthesis of mouse codon-optimized FLP (FLPo) and ΦC31 (ΦC31o) SSRs, which result in recombination efficiencies similar to Cre.

## Introduction

The use of site-specific recombinases (SSRs) both *in vitro* and *in vivo*, have proven to be useful tools in the analysis of gene function. Upon binding to their target recognition sequences, SSRs can induce the deletion, insertion, or inversion of DNA sequences leading to conditional gene inactivation or expression [1]. The first widely used SSR in mammalian cultured cells and animals was the P1 bacteriophage-derived Cre, a member of the λ integrase family that recognizes homotypic 34 bp loxP recognition sites [2], [3]. To date, Cre recombinase remains the most efficient only SSR to efficiently mediate DNA recombination both *in vitro* and *in vivo*. A second SSR from the λ integrase family, FLP from *Saccharomyces cerevisiae*, has also been used in mammals [4] and recognizes distinct 34 bp FRT sites [5]. Initial use of FLP in mammalian cells revealed inefficient recombinase activity due to thermo-instability of the protein [6]. Subsequent screening for thermo-stable mutants resulted in the identification of FLPe, with a 4-fold increase in recombination efficiency [7]. Despite this improvement, the recombination efficiency of FLPe in cells remains quite low, at most a 6%, with mosaic recombination found in almost all ES clones [8]. A third SSR, ΦC31 from *Streptomyces lividans*, also displays activity in mammalian cells [9], [10]. Unlike Cre and FLP, ΦC31 mediates DNA recombination at heterotypic binding sequences known as attB and attP sites [10]. Upon recombination of these target sites, hybrid attL or attR sites are created which are refractive to further recombination, locking the newly formed sequences into place [10], a distinct feature desirable in many molecular applications. Although one report suggests that ΦC31 may be nearly as efficient as Cre in mediating recombination in cultured cells [11], others have indicated more limited success [9] and thus the broad utility of this recombinase as a tool remains to be established. In this manuscript we now report that the *de novo* synthesis of mouse codon-optimized FLP (FLPo) and ΦC31 (ΦC31o) SSRs result in DNA recombination efficiencies similar to that of Cre.

## Results and Discussion

One reason for the observed varying degrees of efficiency of SSRs in mammalian cells may be their non-mammalian origin. Achieving high steady-state expression levels of non-endogenous genes in mammalian systems can be difficult, due to differences in amino acid codon usage, or the presence of cryptic splice acceptor/donor sites since these genes do not normally undergo splicing in the native host. To improve their translational efficiency in mammalian cells, FLPe and ΦC31 recombinases were re-engineered *de novo* according to the native amino acid sequence but with mouse codon usage (Figures S1 & S2). The final ΦC31 and FLP DNA coding sequence optimization was designed using the GeneOptimizer software algorithm (Geneart GmbH, Regensburg, Germany; http://www.geneart.com/). During the optimization process a number of sequence motifs were avoided, including internal TATA-boxes, ribosomal entry sites, stretches of AT- and GC-rich sequence, repeat sequences, RNA secondary structure, and cryptic splice and polyadenylation sites (for review on synthetic gene design see [12]). Additionally, the codon-optimized ΦC31 gene (ΦC31optimized or ΦC31o) was synthesized with a reduced number of CpG dinucleotides, to avoid gene silencing associated with DNA methylation at such sites [13]. Last, the overall base composition of the codon-optimized FLPe gene (FLP optimized or FLPo) was modified to prolong mRNA half-life, since genes with low G/C content often result in less stable mRNAs and low levels of expression. Two stop codons were included in the optimized SSRs to ensure efficient translational termination.

**Figure 1:**
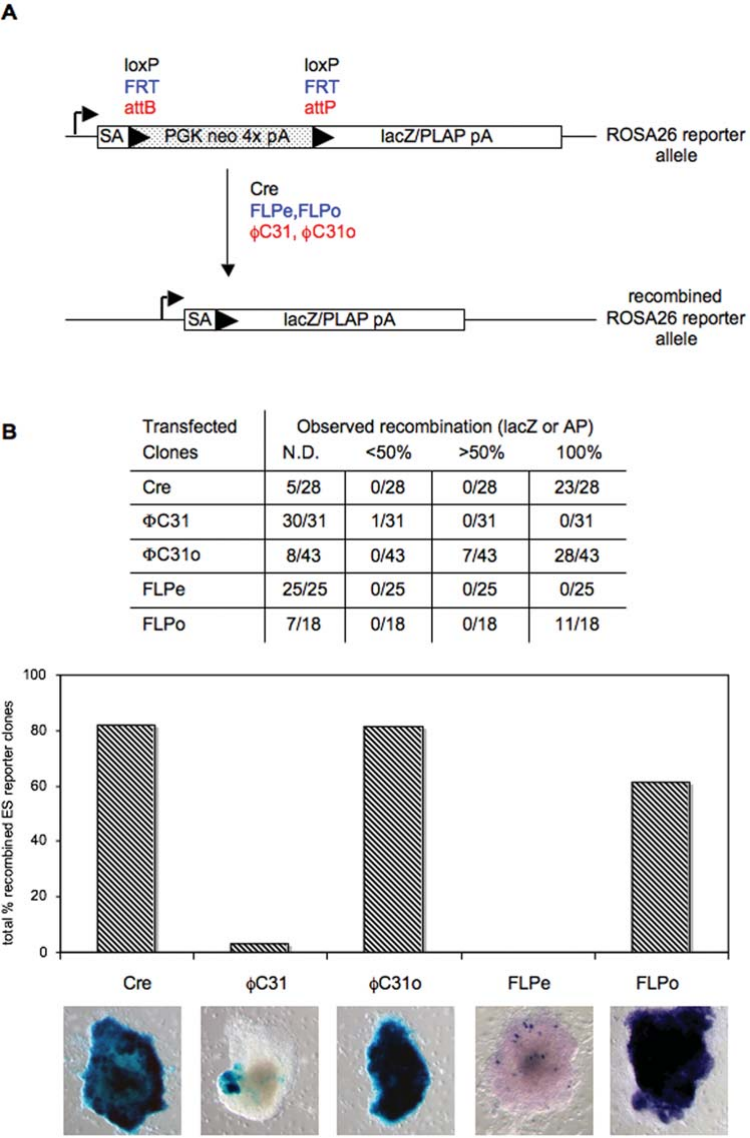
Recombination analysis of SSR activity on stably transfected ES cells. (A) Schematic diagram of SSR reporter assay system. A ΦC31 β-galactosidase reporter ES cell line was constructed at the ROSA26 locus [14], except that the stop cassette was flanked by minimal 35 bp attB and 39 bp attP target DNA recognition sites[10]. These ES cells were used to generate a ΦC31 reporter mouse strain. (B) ES reporter cell lines for Cre [14], FLP [16], and ΦC31 (described within) were stably co-transfected with linearized SSR and PGKHygromycin. SSR-mediated recombination activity was assessed on hygromycin-resistant ES cell colonies by X-gal (Cre and ΦC31) and AP staining (FLP). Variable degrees of recombination were observed in colonies, as judged by the percentage of cells reacting with X-Gal; representative ES cell colonies are shown. N.D.- not detected.

Recombination activity of the re-engineered FLPo and ΦC31o were directly compared in appropriate ROSA26 based ES reporter lines to the native FLPe and ΦC31 sequences, as well as to Cre (Figure 1A). To allow a direct comparison between SSRs, all expression constructs were driven by the phosphoglycerate kinase 1 (PGK) promoter, included a Kozak consensus translational start sequence, an SV40 nuclear localization signal, and were terminated by a bovine growth hormone polyadenylation sequence. Since a multitude of changes were made across the entire coding sequences of the codon-optimized recombinases, any potential observed differences in DNA recombination activity would be due to the combined effects of a number of parameters that were altered in the re-engineering process. Recombination activity for ΦC31 and ΦC31o was assessed in R26attR reporter ES cells that contain a β-galactosidase gene disrupted by an attB and attP-flanked stop cassette (Figure S3).

The results of these stable transfection assays (Figure 1B) indicate that codon-optimization of the FLPe and ΦC31 genes significantly improves recombination activity in ES cells, to a level similar to that observed with Cre recombinase. Analysis of X-gal staining revealed that a majority of ES cell colonies displayed complete Cre-mediated recombination activity when harboring an integrated PGKCre expression vector compared to reporter cell line alone. However, ES cell colonies containing an integrated ΦC31 expression construct showed either no or very mosaic X-Gal staining. In marked contrast, most ES cell clones containing an integrated codon-optimized ΦC31o construct displayed robust X-gal staining. We next performed alkaline phosphatase staining on FLP reporter ES cells and observed no recombination mediated by the native FLPe gene (although using pCAGGS-FLPe we observed ∼5% of clones with mosaic recombination, as previously described [8]). In contrast, the majority of the ES colonies displayed efficient FLP-mediated recombination activity when containing an integrated codon-optimized FLPo expression vector. These results indicate that Cre, FLPo and ΦC31o, all achieve similar recombination efficiency in ES cells.

**Figure 2:**
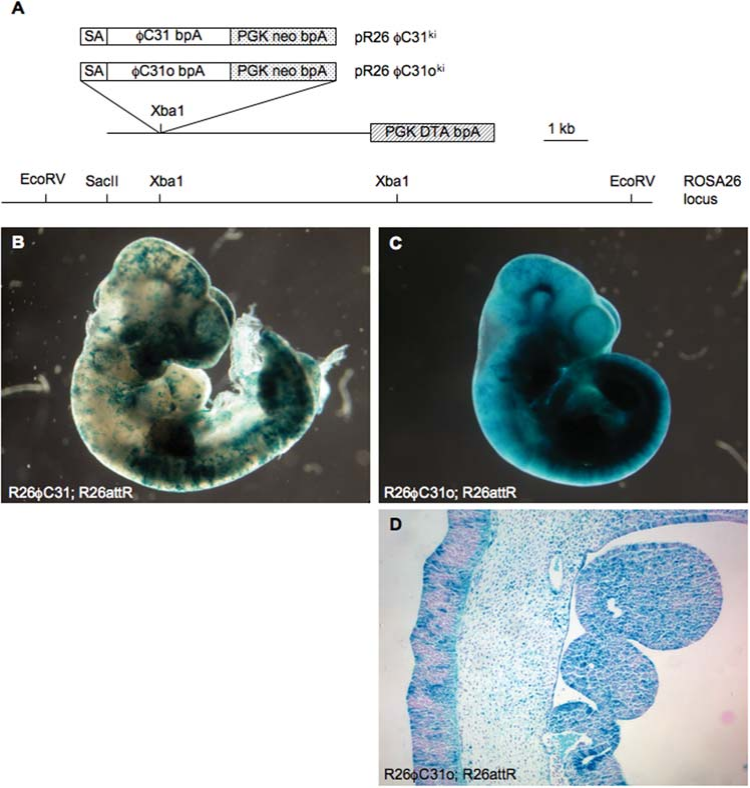
Analysis of R26ΦC31 and R26ΦC31o activity *in vivo.* (A) Diagram of ΦC31 and ΦC31o knock-in vectors targeted to the ROSA26 locus. Whole mount X-Gal staining of E10.5 (B) R26ΦC31; R26attR and (C) R26ΦC31o; R26attR compound heterozygous embryos. (D) Sagittal section of X-Gal stained E10.5 R26ΦC31o; R26attR compound heterozygote.

Based on the promising activity of ΦC31o in mediating recombination events in ES cells, we tested recombination efficiency *in vivo* by targeting the coding sequences of ΦC31 and the codon-optimized ΦC31o to the broadly expressed ROSA26 locus [14] (Figure 2A). Such strains would potentially allow for more complex genetic analysis when used in combination with Cre and FLP expressing mouse strains. Although continued expression of ΦC31 has been shown to lead to chromosomal aberrations in primary human fibroblasts [15], R26ΦC31 and R26ΦC31o heterozygous or homozygous mice were viable and neither showed any obvious deleterious effects from the widespread expression of the SSR. Recombination activity was assessed by crossing R26ΦC31 and R26ΦC31o mice to the R26attR reporter mouse strain. E10.5 embryos carrying the R26attR reporter allele alone exhibited no background X-gal staining (data not shown). R26ΦC31; R26attR compound heterozygotes exhibited a variable low-level mosaic pattern of X-Gal staining (Figure 2B). In contrast, R26ΦC31o; R26attR embryos exhibited broad X-gal staining (Figure 2C), although sectioning (Figure 2D) revealed that recombination was not as efficient as was observed in R26Cre; R26R embryos [14].

The use of multiple, highly efficient SSRs in ES cells and mice now opens possibilities for a broader range of molecular manipulations. These not only include the potential for additional genetic alterations during ES cell expansion (such as removal of selectable markers) and performing multiple general or tissue-specific gene knockouts, but also for studying gene function in over-lapping domains of expression. Additionally, the use of multiple SSRs could potentially be used for controlling gene expression in a temporal “off-on-off” manner in single or multiple cell types or tissues. The optimized ΦC31o and FLPo expression constructs will be made available to academic researchers through the Addgene plasmid repository (http://www.addgene.org/).

## Materials and Methods

### Construction of site-specific recombinase expression vectors

The coding sequence of FLPo and ΦC31o was commercially synthesized *de novo* (Geneart GmbH, Regensburg, Germany) based on the published FLPe and ΦC31 coding sequence [8], [11]. The coding sequence of endogenous ΦC31 was PCR-amplified from phage lysate (Deutsche Sammlung von Mikroorganismen und Zellkulturen (DSMZ) GmbH, Braunschweig, Germany). A C-terminal SV40 nuclear localization signal was also added to the endogenous ΦC31 coding sequence as previously described [11]. The FLPo, ΦC31o, and ΦC31 coding sequences were blunt cloned into mammalian expression vectors driven by the high expressing phosphoglycerate kinase 1 (PGK) promoter and containing the bovine growth hormone polyadenylation (bpA) sequence.

### Reporter constructs and mice

ES cells harboring a Cre-inducible β-galactosidase or FLP-inducible PLAP reporter gene integrated at the broadly-expressed ROSA26 locus were used as previously described to monitor Cre or FLP activity [14], [16]. A ΦC31/att β-galactosidase reporter ES cell line was constructed similarly to the above, except that the stop cassette was flanked by the minimal target DNA recognition sites, the 35 bp attB (GGTGCCAGGGCGTGCCCTTGGGCTCCCCGGGCGCG) and the 39 bp attP (CCCCAACTGGGGTAACCTTTGAGTTCTCTCAGTTGGGGG) [10]. These ES cells were used to generate a ΦC31 reporter mouse strain gene to monitor ΦC31 and ΦC31o DNA recombinase activity *in vivo*. Similarly, the ΦC31o and ΦC31 coding sequences were blunt cloned into the pROSA26-1 vector to generate mouse strains that broadly express this SSR.

### ES cell transfection assay

20 µg of each linearized SSR expression construct was co-electroporated in a 10∶1 molar ratio with PGKHygromycin into corresponding ES reporter cell lines for Cre [14], FLP [16], and ΦC31 (described within). After 10 days of antibiotic selection, Hygromycin-resistant ES clones were PCR genotyped for the presence of the SSR expression constructs and SSR-mediated DNA recombination was assessed by AP and X-gal staining.

### X-gal and AP staining

Alkaline phosphatase (FLP) and β-galactosidase (Cre and φC31) activity in ES cells and embryos was visualized as previously described [14], [16].

## Supporting Information

Figure S1 Nucleotide sequence of FLPo. A mouse codon-optimized FLP gene containing an N-terminal SV40 nuclear localization signal was generated de novo (GENEART AG, Regensburg, Germany) according to previously described FLPe amino acid sequence [8].
(0.03 MB DOC)

Figure S2 Nucleotide sequence of ΦC31o. A mouse codon-optimized ΦC31 gene with C-terminal SV40 nuclear localization signal was synthesized de novo according to the native ΦC31 amino acid sequence (GENEART AG, Regensburg, Germany).
(0.04 MB DOC)

Figure S3 Establishment of ROSAattR reporter line. Diagram of ϕC31 reporter knock-in vector targeted to the ROSA26 locus. The stop cassette is flanked by 35 bp attB and 39 bp attP sites, as previously described [10].
(0.06 MB TIF)
