# Supplementary material for: High-Efficiency FLP and ΦC31 Site-Specific Recombination in Mammalian Cells
Source: PLoS One. 2007 Jan 17;2(1):e162. doi: 10.1371/journal.pone.0000162 (PMC1764711; doi:10.1371/journal.pone.0000162)
Supplement: Figure S1 — Nucleotide sequence of FLPo. A mouse codon-optimized FLP gene containing an N-terminal SV40 nuclear localization signal was generated de novo (GENEART AG, Regensburg, Germany) according to previously described FLPe amino acid sequence [8]. (0.03 MB DOC) [file pone.0000162.s001.doc]

1 ATG GCT CCT AAG AAG AAG AGG AAG GTG ATG AGC CAG TTC GAC ATC CTG TGC AAG ACC CCC CCC AAG GTG CTG GTG 75

1 M A P K K K R K V M S Q F D I L C K T P P K V L V 25

76 CGG CAG TTC GTG GAG AGA TTC GAG AGG CCC AGC GGC GAG AAG ATC GCC AGC TGT GCC GCC GAG CTG ACC TAC CTG 150

26 R Q F V E R F E R P S G E K I A S C A A E L T Y L 50

151 TGC TGG ATG ATC ACC CAC AAC GGC ACC GCC ATC AAG AGG GCC ACC TTC ATG AGC TAC AAC ACC ATC ATC AGC AAC 225

51 C W M I T H N G T A I K R A T F M S Y N T I I S N 75

226 AGC CTG AGC TTC GAC ATC GTG AAC AAG AGC CTG CAG TTC AAG TAC AAG ACC CAG AAG GCC ACC ATC CTG GAG GCC 300

76 S L S F D I V N K S L Q F K Y K T Q K A T I L E A 100

301 AGC CTG AAG AAG CTG ATC CCC GCC TGG GAG TTC ACC ATC ATC CCT TAC AAC GGC CAG AAG CAC CAG AGC GAC ATC 375

101 S L K K L I P A W E F T I I P Y N G Q K H Q S D I 125

376 ACC GAC ATC GTG TCC AGC CTG CAG CTG CAG TTC GAG AGC AGC GAG GAG GCC GAC AAG GGC AAC AGC CAC AGC AAG 450

126 T D I V S S L Q L Q F E S S E E A D K G N S H S K 150

451 AAG ATG CTG AAG GCC CTG CTG TCC GAG GGC GAG AGC ATC TGG GAG ATC ACC GAG AAG ATC CTG AAC AGC TTC GAG 525

151 K M L K A L L S E G E S I W E I T E K I L N S F E 175

526 TAC ACC AGC AGG TTC ACC AAG ACC AAG ACC CTG TAC CAG TTC CTG TTC CTG GCC ACA TTC ATC AAC TGC GGC AGG 600

176 Y T S R F T K T K T L Y Q F L F L A T F I N C G R 200

601 TTC AGC GAC ATC AAG AAC GTG GAC CCC AAG AGC TTC AAG CTG GTG CAG AAC AAG TAC CTG GGC GTG ATC ATT CAG 675

201 F S D I K N V D P K S F K L V Q N K Y L G V I I Q 225

676 TGC CTG GTG ACC GAG ACC AAG ACA AGC GTG TCC AGG CAC ATC TAC TTT TTC AGC GCC AGA GGC AGG ATC GAC CCC 750

226 C L V T E T K T S V S R H I Y F F S A R G R I D P 250

751 CTG GTG TAC CTG GAC GAG TTC CTG AGG AAC AGC GAG CCC GTG CTG AAG AGA GTG AAC AGG ACC GGC AAC AGC AGC 825

251 L V Y L D E F L R N S E P V L K R V N R T G N S S 275

826 AGC AAC AAG CAG GAG TAC CAG CTG CTG AAG GAC AAC CTG GTG CGC AGC TAC AAC AAG GCC CTG AAG AAG AAC GCC 900

276 S N K Q E Y Q L L K D N L V R S Y N K A L K K N A 300

901 CCC TAC CCC ATC TTC GCT ATC AAG AAC GGC CCT AAG AGC CAC ATC GGC AGG CAC CTG ATG ACC AGC TTT CTG AGC 975

301 P Y P I F A I K N G P K S H I G R H L M T S F L S 325

976 ATG AAG GGC CTG ACC GAG CTG ACA AAC GTG GTG GGC AAC TGG AGC GAC AAG AGG GCC TCC GCC GTG GCC AGG ACC 1050

326 M K G L T E L T N V V G N W S D K R A S A V A R T 350

1051 ACC TAC ACC CAC CAG ATC ACC GCC ATC CCC GAC CAC TAC TTC GCC CTG GTG TCC AGG TAC TAC GCC TAC GAC CCC 1125

351 T Y T H Q I T A I P D H Y F A L V S R Y Y A Y D P 375

1126 ATC AGC AAG GAG ATG ATC GCC CTG AAG GAC GAG ACC AAC CCC ATC GAG GAG TGG CAG CAC ATC GAG CAG CTG AAG 1200

376 I S K E M I A L K D E T N P I E E W Q H I E Q L K 400

1201 GGC AGC GCC GAG GGC AGC ATC AGA TAC CCC GCC TGG AAC GGC ATC ATC AGC CAG GAG GTG CTG GAC TAC CTG AGC 1275

401 G S A E G S I R Y P A W N G I I S Q E V L D Y L S 425

1276 AGC TAC ATC AAC AGG CGG ATC TGA 1299

426 S Y I N R R I * 433
