# Supplementary material for: High-Efficiency FLP and ΦC31 Site-Specific Recombination in Mammalian Cells
Source: PLoS One. 2007 Jan 17;2(1):e162. doi: 10.1371/journal.pone.0000162 (PMC1764711; doi:10.1371/journal.pone.0000162)
Supplement: Figure S2 — Nucleotide sequence of ΦC31o. A mouse codon-optimized ΦC31 gene with C-terminal SV40 nuclear localization signal was synthesized de novo according to the native ΦC31 amino acid sequence (GENEART AG, Regensburg, Germany). (0.04 MB DOC) [file pone.0000162.s002.doc]

1 ATG GAT ACC TAC GCC GGA GCC TAC GAC AGA CAG AGC CGG GAG AGA GAG AAC AGC AGC GCC GCC AGC CCC GCC ACC 75

1 M D T Y A G A Y D R Q S R E R E N S S A A S P A T 25

76 CAG AGA AGC GCC AAC GAG GAT AAG GCC GCC GAT CTG CAG AGA GAG GTG GAG AGG GAC GGC GGC AGA TTC AGA TTT 150

26 Q R S A N E D K A A D L Q R E V E R D G G R F R F 50

151 GTG GGC CAC TTC AGC GAG GCC CCT GGC ACC AGC GCC TTC GGC ACC GCC GAG AGA CCC GAG TTC GAG AGA ATC CTG 225

51 V G H F S E A P G T S A F G T A E R P E F E R I L 75

226 AAC GAG TGT AGG GCC GGC AGG CTG AAC ATG ATC ATC GTG TAC GAC GTG TCC CGG TTC AGC AGG CTG AAG GTG ATG 300

76 N E C R A G R L N M I I V Y D V S R F S R L K V M 100

301 GAC GCC ATC CCT ATC GTG TCC GAG CTG CTG GCC CTG GGC GTG ACC ATC GTG TCC ACC CAG GAA GGC GTC TTT AGA 375

101 D A I P I V S E L L A L G V T I V S T Q E G V F R 125

376 CAG GGC AAC GTG ATG GAC CTG ATC CAC CTG ATC ATG AGG CTG GAC GCC AGC CAC AAG GAG AGC AGC CTG AAG AGC 450

126 Q G N V M D L I H L I M R L D A S H K E S S L K S 150

451 GCC AAG ATC CTG GAC ACC AAG AAC CTG CAG AGG GAG CTG GGC GGC TAT GTG GGC GGC AAG GCC CCC TAC GGC TTC 525

151 A K I L D T K N L Q R E L G G Y V G G K A P Y G F 175

526 GAG CTG GTG TCC GAG ACC AAG GAG ATC ACC CGG AAC GGC AGG ATG GTG AAC GTG GTG ATC AAC AAG CTG GCC CAC 600

176 E L V S E T K E I T R N G R M V N V V I N K L A H 200

601 AGC ACC ACC CCC CTG ACC GGC CCC TTC GAG TTT GAG CCC GAC GTG ATC AGG TGG TGG TGG CGG GAG ATC AAG ACC 675

201 S T T P L T G P F E F E P D V I R W W W R E I K T 225

676 CAC AAG CAC CTG CCT TTC AAG CCC GGC AGC CAG GCC GCC ATC CAC CCC GGC AGC ATC ACC GGC CTG TGT AAG AGA 750

226 H K H L P F K P G S Q A A I H P G S I T G L C K R 250

751 ATG GAC GCC GAC GCC GTG CCC ACC AGA GGC GAG ACC ATC GGC AAG AAA ACC GCC AGC AGC GCC TGG GAC CCC GCC 825

251 M D A D A V P T R G E T I G K K T A S S A W D P A 275

826 ACC GTG ATG AGA ATC CTG AGG GAC CCT AGG ATC GCC GGC TTC GCC GCC GAG GTG ATC TAC AAG AAG AAG CCC GAC 900

276 T V M R I L R D P R I A G F A A E V I Y K K K P D 300

901 GGC ACC CCC ACC ACC AAG ATC GAG GGC TAC AGA ATC CAG AGA GAC CCC ATC ACC CTG AGA CCT GTG GAG CTG GAC 975

301 G T P T T K I E G Y R I Q R D P I T L R P V E L D 325

976 TGT GGC CCT ATC ATC GAG CCT GCC GAG TGG TAC GAG CTG CAG GCC TGG CTG GAC GGC AGA GGC AGA GGC AAG GGC 1050

326 C G P I I E P A E W Y E L Q A W L D G R G R G K G 350

1051 CTG AGC AGA GGC CAG GCC ATC CTG AGC GCC ATG GAC AAG CTG TAC TGT GAG TGT GGC GCC GTG ATG ACC AGC AAG 1125

351 L S R G Q A I L S A M D K L Y C E C G A V M T S K 375

1126 AGA GGC GAG GAG AGC ATC AAG GAC AGC TAC CGG TGC CGG AGA AGA AAG GTG GTG GAC CCC AGC GCC CCT GGC CAG 1200

376 R G E E S I K D S Y R C R R R K V V D P S A P G Q 400

1201 CAC GAG GGC ACC TGT AAT GTG AGC ATG GCC GCC CTG GAC AAG TTC GTG GCC GAG CGG ATC TTC AAC AAG ATC CGG 1275

401 H E G T C N V S M A A L D K F V A E R I F N K I R 425

1276 CAC GCC GAG GGC GAC GAG GAG ACC CTG GCC CTG CTG TGG GAG GCC GCC AGA AGA TTC GGC AAG CTG ACC GAG GCC 1350

426 H A E G D E E T L A L L W E A A R R F G K L T E A 450

1351 CCC GAG AAG AGC GGC GAG AGG GCC AAC CTG GTG GCC GAG AGA GCC GAC GCC CTG AAC GCC CTG GAG GAG CTG TAC 1425

451 P E K S G E R A N L V A E R A D A L N A L E E L Y 475

1426 GAG GAC AGA GCC GCC GGA GCC TAT GAC GGC CCT GTG GGC AGG AAG CAC TTC AGA AAG CAG CAG GCC GCC CTG ACC 1500

476 E D R A A G A Y D G P V G R K H F R K Q Q A A L T 500

1501 CTG AGA CAG CAG GGC GCC GAG GAA AGA CTG GCC GAG CTG GAG GCC GCC GAG GCC CCT AAG CTG CCC CTG GAT CAG 1575

501 L R Q Q G A E E R L A E L E A A E A P K L P L D Q 525

1576 TGG TTC CCC GAG GAT GCC GAC GCC GAC CCC ACC GGC CCC AAG TCC TGG TGG GGC AGA GCC AGC GTG GAC GAC AAG 1650

526 W F P E D A D A D P T G P K S W W G R A S V D D K 550

1651 AGG GTG TTC GTG GGC CTG TTC GTG GAT AAG ATC GTG GTG ACC AAG AGC ACC ACC GGC AGG GGC CAG GGC ACC CCC 1725

551 R V F V G L F V D K I V V T K S T T G R G Q G T P 575

1726 ATC GAG AAG AGA GCC AGC ATC ACC TGG GCC AAG CCT CCC ACC GAC GAC GAC GAG GAT GAC GCC CAG GAC GGC ACC 1800

576 I E K R A S I T W A K P P T D D D E D D A Q D G T 600

1801 GAG GAC GTG GCC GCC CCT AAG AAA AAG CGG AAA GTG TGA 1839

601 E D V A A P K K K R K V * 613
