# Supplementary figures and images for: High-Efficiency FLP and ΦC31 Site-Specific Recombination in Mammalian Cells
Source: PLoS One. 2007 Jan 17;2(1):e162. doi: 10.1371/journal.pone.0000162 (PMC1764711; doi:10.1371/journal.pone.0000162)

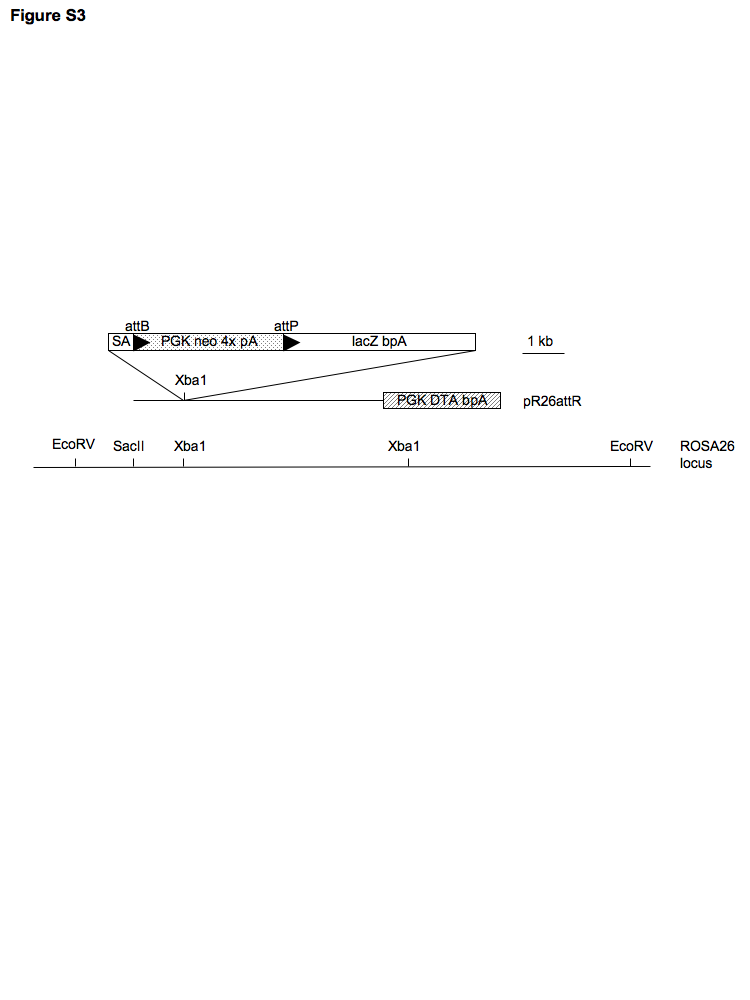

Supplement: Figure S3 — Establishment of ROSAattR reporter line. Diagram of ϕC31 reporter knock-in vector targeted to the ROSA26 locus. The stop cassette is flanked by 35 bp attB and 39 bp attP sites, as previously described [10]. (0.06 MB TIF) [file pone.0000162.s003.tif]
